# The structural-demographic theory revisited: An empirical test for industrialized societies

**DOI:** 10.1371/journal.pone.0287912

**Published:** 2023-11-02

**Authors:** Oana-Maria Georgescu

**Affiliations:** European Central Bank, Frankfurt am Main, Germany; Centre for Studies in Social Sciences, Calcutta, INDIA

## Abstract

The structural demographic theory for industrialized societies generates three testable predictions. The first prediction is that labor oversupply leads to declining (relative) wages. The second prediction is that labor oversupply leads to elite overproduction: as relative wages fall, elite incomes display a hump-shaped pattern while elite numbers increase. The third prediction is that elite overproduction leads to political instability. I test these predictions on US data by combining evidence from existing studies with empirical proxies for elite numbers and elite income. The predictions are not supported by the data. First, labor oversupply cannot explain the polarization of wages and the decline in relative wages. The largest share in wage variance is explained by automation. Second, the data shows that as relative wages fall, elite incomes increase, in contrast to the hump-shaped pattern displayed by the model. Third, elite overproduction did not predict political instability in the last decades. Political instability is modelled by the Political Stress Index (PSI). The reviewed evidence shows that the increase in the model based PSI in the last decades is driven by the increase in inequality. The rise in inequality was caused by technological change, globalisation and to a lesser extent by the erosion of labor market institutions. Attributing the rise in inequality and the resulting increase in political instability to labor oversupply rather than to the lost race between education and technology may weaken incentives to design effective policies addressing the inefficiencies in the US education system.

## Introduction

A proper understanding of the drivers of political instability and inequality is essential for the design of effective policies targeting the underlying structural causes. The structural-demographic theory (SDT) posits that human societies can be viewed as complex systems in which non-linear feedback loops between the three main pillars of society—the general population, the elites and the state—can lead to political instability. The intuition is as follows: as population grows, wages fall and the return on capital (land) increases. These growth opportunities are associated with higher upward social mobility. Population growth and high upward social mobility lead to an increase in intra-elite competition. At the same time, life quality deteriorates for the ‘masses’ due to falling wages. Political instability emerges as polarized elites mobilize dissatisfied masses to their own benefit, often on the background of strained public finances.

The causal network of the structural-demographic theory (SDT) is translated into three central predictions for industrialized societies. These predictions derive from the “labor oversupply principle”, the “elite overproduction principle” and the “instability principle” (see [1], p. 17). The model outlined in [1, 2] tests these predictions on data for the United States for the period between 1948 and 2020. The evidence reviewed hereafter shows that these predictions are not supported by the data.

The first prediction is that labor oversupply leads to declining (relative) wages. Evidence from micro-data overwhelmingly contradicts the prediction that labor oversupply drives down real and relative wages in the US in the period between 1980 and 2016. As I explain below, relative wages and inequality are conceptually equivalent, in the sense that a decrease in relative wages can be interpreted as an increase in inequality. The empirical evidence suggests that inequality increases (i.e. relative wages decrease) due to a complex interaction between technology and labor supply across different demographic groups. This evidence is inconsistent with a simple story of labor oversupply driving the decrease in relative wages as implied by the SDT.

The second prediction is that labor oversupply leads to elite overproduction. As relative wages fall, elite incomes display a hump-shaped pattern while elite numbers increase [1], p. 23. To test this prediction, I use empirical proxies for elite numbers and elite income. I find that the pattern of elite incomes is not consistent with the predictions made by the theory: as relative wages fall, elite incomes increase, in contrast to the hump-shaped pattern displayed by the model-based elite income.

The third prediction is that elite overproduction leads to political instability. Political instability is quantified by the Political Stress Index (PSI) as in [2]. The latter is a function of the mass mobilisation potential, the elite mobilisation potential, state fiscal distress and trust in political institutions. The first two variables are key for testing the predictions implied by the structural-demographic theory. The increase in both variables is driven by the relative wage, a proxy variable for inequality.

The fact that relative wage and inequality are equivalent matters for two reasons. First, it changes the interpretation of the recent increase in the Political Stress Indicator: it is easy to see that the increase in the model based Political Stress Indicator in the last three decades is driven by rising inequality. Second, the reviewed evidence shows that the rise in inequality is caused by technological change, globalisation and to a lesser extent by the erosion of labor market institutions. The above evidence has implications for the validity of the central predictions of the structural-demographic theory for industrialized societies. These implications are discussed in the next sections. The main take-away is that, while the structural-demographic theory receives empirical support for pre-industrial societies [3], its predictions for industrialized societies are not supported by the data.

## Material and methods

### Predictions

I rely on existing empirical evidence as well as empirical proxies for elite incomes and elite numbers to formulate three testable predictions implied by the structural demographic theory:

1. Labor oversupply explains the variance in real wages and the decline in relative wages.
2. As relative wages fall, elite incomes display a hump-shaped pattern while elite numbers increase.
3. Elite overproduction leads to a steep increase in the Political Stress Indicator and thus predicts political instability.

I test these predictions on US data over the period 1940–2016.

### Data

#### Labor supply and wages

Data on the variance decomposition of US wages in the period between 1980 and 2016 was obtained from table A.III, columns (1) and (3) in the Supplementary Material in [4]. The table reports the share of variance explained by labor supply, education and automation.

#### Elite numbers

The first proxy of elite numbers is defined as the share of households with income larger than $150,000 expressed in 2020 dollars. The data was obtained from Table A-2, “Households by Total Money Income, Race, and Hispanic Origin of Householder: 1967 to 2020”. The data is available under 2020 Census Tables, Table A-2.

#### Elite incomes

The first proxy for elite incomes is the share of national income of households with incomes larger than the 80^*th*^ percentile of the US income distribution. The data was obtained from the 2021 US Census, Table A4-b, “Selected Measures of Household Income Dispersion: 1967 to 2020”. The data is available under 2020 Census Tables, Table A4.

The second proxy for elite income is based on the US law school graduates income. The data was obtained from the National Association for Law Placement. The data is available under NALP Law Graduates Data. Income per graduates decreased after the 2008 crisis, reaching 75% of the pre-crisis level in 2011, increasing steadily thereafter, reaching 154% of the pre-crisis level in 2019.

The second proxy for elite numbers is defined analogous as in [1] as the number of enrolled law students relative to the total US population multiplied by 10,000. The data was obtained from the Law School Admission Council. Data is available under Law School Enrollment. Data on the US population was obtained from the Federal Reserve Bank of St. Louis., under the code “POPTOTUSA647NWDB”.

#### Political Stress Indicator

I compute the Political Stress Indicator (PSI) as the product of the mass-mobilisation potential (MMP), the elite mobilisation potential (EMP) and state fiscal distress (SFD), as in [1, 2], equation 13.2:

$$\begin{array}{c} PSI_{model,t}=MMP\cdot EMP\cdot SFD \end{array}$$ (1)

Considering the definition of each of the three components above, the PSI can be rewritten as in [1, 2], equation 13.2 as:

$$\begin{array}{c} PSI_{model,t}=w_{rel,t}^{-1}\cdot N_{urb,t}\cdot N_{20\_29,t}\cdot \epsilon_{t}^{-1}\cdot e_{t}\cdot D_{t}\cdot \left(1-T_{t}\right) \end{array}$$ (2)

Where *w*_*rel*_ refers to the relative wage, defined as the ratio of median wage to GDP per capita, *e* and *ϵ* to elite numbers and elite income respectively; *D* is the ratio of public debt to GDP, *N*_*urb*_ the urbanisation rate, *N*_20_29_ the share of the population aged 20 to 29 and *T* is the share of the population that trusts the government. The data for these variables was obtained from [2].The first three terms in Eq 2, $w_{rel,t}^{-1}$, *N*_*urb*,*t*_ and *N*_20_29,*t*_, capture the mass-mobilisation potential. The middle two terms, *ϵ*^−1^ and *e*_*t*_, capture the elite mobilisation potential. The last two terms, *D*_*t*_ and (1 − *T*_*t*_) capture state fiscal distress.

Fig 3 compares the model implied PSI reported in [2] with the PSI derived by replacing the model implied *ϵ*_*t*_ and *e* in Eq 2 with their empirical proxies. To see whether the variables included in the model based PSI can predict political instability, I run a regression having demonstrations in the US as a dependant variable and the variables included in the model based PSI as predictors. Results are shown in S1 Table. The sample period is from 1946 to 2018. The data was obtained from [2]. The regression was run in Stata.

## Labor oversupply principle

The labor oversupply principle states that “when the supply of labor exceeds its demand, the price of labor decreases” [1], p. 17. In turn, declining wages lead to popular discontent and therefore an increase in the mass mobilisation potential. The labor oversupply principle reflects a basic economic concept: when supply goes up, the price goes down. The question is whether labor oversupply can explain the dynamics of real and relative wages in industrialized societies in general and in particular in the US in the last decades.

The empirical illustration of the labor oversupply principle is the mass mobilisation potential (MMP) [1, 2, 5]. As shown in Eq 2, the mass mobilisation potential is used as input for the calculation of the Political Stress Indicator. The MMP depends on the inverse of the relative wage, the size of the urban population and the share of the cohorts aged between 20 and 29 in the total population (‘youth bulges’). Two observations emerge from the visual inspection of these variables. First, the share of the population aged between 20 and 29 years has been stagnating in the United States in the last two decades (see S1 Fig). This pattern is noteworthy given that the Political Stress Indicator is increasing in the size of the ‘youth bulges’. Second, the steady path of the share of the urban population stands in contrast to the cyclical pattern of the relative wage and the Political Stress Indicator, suggesting that the relative wage is a major driver behind the recent increase in the Political Stress Indicator, (see also Fig 3.4 in [1] and S2 Fig for the long-run dynamics of the relative wage).

### Relative wages and inequality

Relative wages are defined as the ratio of median wages to GDP per capita, conceptually corresponding to what the economists call the labor share in total output. Similarly, the labor share is measured as the ratio of total wages to total output, (see Estimating the U.S. labor share).The interpretation of the labor share given by the US Bureau of Labor Statistics is the following: “The labor share is an indicator of the extent to which workers share in the economy’s output.” This is also the sense in which the relative wage is used for the calculation of the mass mobilisation potential: “The question is: what proportion of economic growth translates into increased incomes for workers, and how does this quantity change with time?” [1], p. 66.

Researchers and policy makers often interpret the labor share as a measure of inequality. This interpretation has two caveats: first, the numerator of the labor share refers to total wages. Total wages also include top wages, which leads to the underestimation of inequality, (see also [6] for a discussion on the statistical artifacts affecting the labor share). Using median wages or a different percentile of the income distribution would be more informative. Second, the denominator of the labor share, total output, can also vary due to changes in capital (depreciation) and net foreign income. Gross national income, which is defined as output net of capital costs and net foreign income is better suited as a denominator of the labor share. To account for these caveats, inequality researchers often define inequality as the share of national income held by the bottom 50% earners divided by the gross national income [7, 8]. Since median wages are used in the numerator of relative wages in [1, 2], relative wages are more closely related to the inequality measure typically used in the literature than the labor share, see also S3 Fig. The fact that GDP per capita is used instead of total GDP just reflects consistency with the numerator—i.e the median wage. This does not change the logical interpretation of relative wage as a proxy measure of inequality.

Since the relative wage measures inequality and relative wages are the key determinant of the dynamics of the Political Stress Indicator in [2], it follows that the increase in the latter is driven by wage inequality. Thus, the predictions of the structural-demographic theory must be discussed in conjunction with the drivers of wage inequality. As I will argue below, technological change is key in explaining the latter.

### Drivers of US wage inequality

The specification of the mass mobilisation potential in [2] builds on the real wage model outlined in [5]. Consistent with one of the central predictions of the SDT for industrialized societies, the model identifies labor oversupply as the core factor explaining the trend reversal in the real wage starting in 1980’s in the US, in particular the wage of the median male worker (see S4 Fig and [1], p. 94). This conclusion is at odds with the abundant empirical and theoretical evidence emphasizing the essential role played by technology and globalisation in understanding the wage dynamics in the US in the last three decades.

While labor supply can and does exert a negative influence on wages, the negative wage impact is constrained by the response of technology [4]. The relevant parameter in this context is the degree of substitution between labor (workers) and capital (machines, robots, computers). If labor and capital are substitutes, then machines replace people and wages decline. If instead labor and capital are complements, then machines increase the productivity of people and wages increase. The consensus among labor economists is that labor and capital were mostly complementary in the last century, depending also on the type of task performed by each worker [4, 9–11]. The important insight is that technology need not be exogenous, but can be endogenous to the labor supply. Thus, to understand wage dynamics one needs to understand the ambiguous impact of technology on wages in response to a change in the size and the composition of labor supply.

A compelling explanation for the recent widening in wage inequality in the US is the automation induced increase in the demand for non-routine tasks at the expense of demand for routine tasks, leading to a polarization in employment [4, 12, 13]. [12] draw a distinction between non-routine and routine tasks. Both of these tasks can be either cognitive or manual. Cognitive non-routine tasks involving problem-solving, judgement and creativity are required in managerial, technical and professional occupations. Manual non-routine tasks are those that require flexibility and physical adaptability, such as personal care, food and cleaning service as well as protective service. Cognitive and manual routine tasks are required in occupations such as sales, administrative, production and operator.

Between 1979 and 2009, the employment share of occupations intensive in non-routine cognitive and manual tasks increased, while the employment share of routine cognitive and manual tasks decreased, see [12] and S5 Fig. The explanation is that middle-skilled jobs involving routine tasks such as the organisation, storage, retrieval of information or assembly of parts can be performed by computers or machines. In contrast, non-routine tasks involving problem solving or physical flexibility cannot (yet) be replaced by machines and computers. For instance, US commuting zones which were more specialized in routine tasks in 1980 showed a higher increase in investment in computers in the period between 1980 and 2006 compared to regions with a lower share of non-routine tasks. The former regions subsequently experienced a larger relative fall in the employment share of routine tasks and higher wage polarization compared to regions less intensive in routine tasks [14].

**Fig 1:**
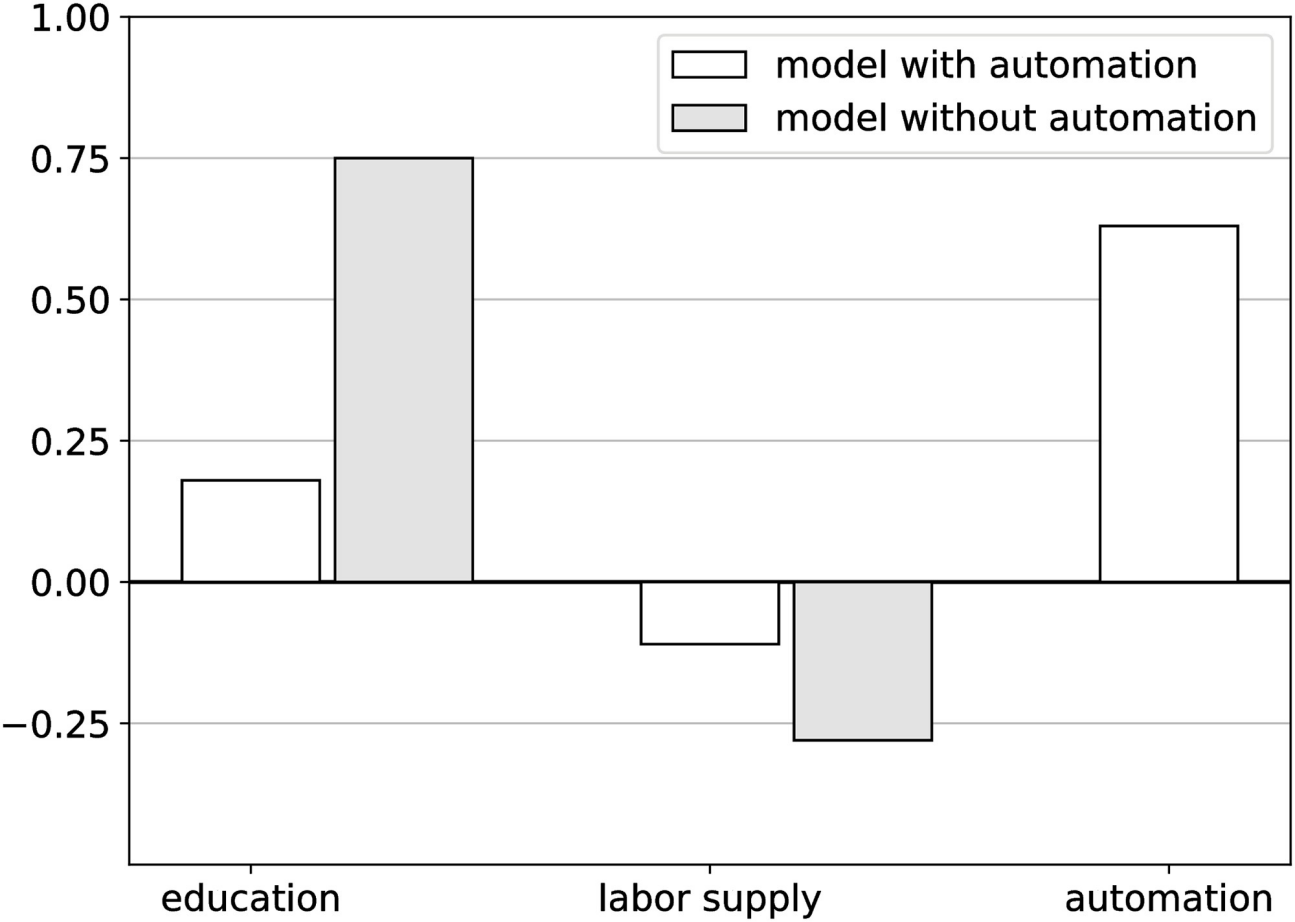
Dynamics of real wages between 1980 and 2016. Variance explained by education, labor supply and automation. Model estimates obtained from table A.III, Supplementary Material [4]. The figure shows the share of variance in real wages that is explained by education, automation and labor supply over the period 1980 to 2016 across 500 demographic groups. Demographic groups are defined by gender, education, age, race, and native/immigrant status. The grey bars show the variance explained by the model specification that does not account for automation. The white bars show the variance of real wages from the model accounting for automation.

These structural changes in the economy had a profound impact on wage dynamics in the last decades. Task displacement due to automation explains around 50% in the decline in the US labor share—i.e. relative wages in [2]—between 1980 and 2016 [4]. Furthermore, Fig 1 shows that 63% of the variance in wages in the period between 1980 and 2016 is accounted for by automation, with labor supply explaining only around 11% of the variance. Once automation is accounted for, the share in wage variance explained by education drops from 75% to around 18%, suggesting that education mediates the impact of automation on wages within each demographic group.

The second important factor affecting the wage structure in the US in the last decades is globalisation. An inflection point is the year 2001, when China joined the World Trade Organisation [15]. Off-shoring of manufacturing jobs to China resulted in the loss of 2–2.5 million jobs in manufacturing in the US [16]. Global trade also depressed wages of existing manufacturing jobs in the US: industries facing the largest competition from China experienced the largest decline in payroll share [6, 17].

Low-educated individuals were the most affected by the trade and automation-induced fall in wages. The higher relative demand for high-skilled workers performing non-routine cognitive tasks compared to low-skilled workers performing non-routine manual tasks lead to an increase in the wage of workers performing the former tasks, while the wage of workers performing the latter tasks decreased (S6 Fig). Since low-educated workers in general perform routine tasks (S7 Fig), the decrease in demand for unskilled labor due to task replacement or off-shoring depresses their earnings. Between 1980 and 2013, real earnings of males with a high school degree decreased by 11%. For high school dropouts, the decrease in real wages amounted to 22%. As a result, the earnings gap between the median college-educated worker and the median high-school educated worker almost doubled in the last thirty years [4, 15, 17]. These trends reflect the complex interaction of technology and labor supply across different demographic groups and are inconsistent with a simple story of labor oversupply proposed by [1, 2, 5].

The wage model testing the predictions of the structural-demographic theory for industrialized societies emphasizes the role of labor market institutions (see [1, 5]). It seems plausible that a stronger role for labor market institutions such as labor unions and a higher minimum wage could have buffered the impact of globalisation and automation on the wage of unskilled workers, redirecting some of the surplus at the top of the income distribution to the bottom earners. Indeed, in the United States union membership declined from 24% in 1973 to 7% in 2016. Furthermore, in 2011 the minimum wage was 24% below its peak in 1960 [15]. Looking across advanced economies, the erosion of labor market institutions is associated with a rise in income inequality at the top of the distribution (see [18]). While union membership is indeed negatively associated with median wage growth, the impact of the decline in union membership on wages is very small after controlling for task displacement due to automation (see [4, 19]), explaining around one tenth of the increase in the Gini coefficient in the US since 1968 [20].

The arguments presented in this section suggest that the first prediction of the structural-demographic theory is not supported by the data. The theory cannot explain the decrease in relative wages and the increase in the mass mobilisation potential over the last decades in the US for two reasons. First, the increase in the mass mobilisation potential is driven by the increase in wage inequality. Second, the downward pressure exerted by the increase in labor supply does not explain the stagnation in real wages and the decline in relative wages starting in the late 1970s. The reviewed shows that the stagnation and polarization in real wages—and the resulting decline in relative wages—are explained by a combination of task-based replacement due to automation, skill-biased technological change, globalization and to a lesser extent by the erosion of labor market institutions. As I will show below, these factors also affect the predictions regarding the elite mobilisation potential.

## Elite overproduction principle

Elites play a central role in the narrative of the structural-demographic theory. The latter predicts that labor oversupply leads to elite overproduction. “Elite overproduction results when elite numbers and appetites exceed the ability of the society to sustain them, leading to spiraling intra-elite competition and conflict” [1], p. 17. Furthermore, “elite overproduction reduces average elite incomes and increases intra-elite competition” [1], p. 23. These definitions suggest that elite overproduction is characterized by increasing elite numbers, falling elite incomes and an increase in intra-elite competition.

Elite overproduction is quantified by the elite mobilisation potential in [2]. The latter is a function of elite numbers and incomes. Both variables are an algebraic manipulation of the relative wage. Model based elite incomes and numbers are derived starting from an arbitrary constant and this number increases at a rate determined by relative wage growth, (see line 59 of the R code “PSI2020” in the Supplementary Material in [2]). The same principle was applied for the calculation of the elite mobilisation potential in [1], p. 32. The external validity of the variables quantifying elite overproduction is essential given that the steep rise in elite numbers in conjunction with the decrease in elite incomes in the last decades is a major contributor to the increase in the Political Stress Indicator (see figure S2 Fig). Nevertheless, no empirical data is used to assess the plausibility of the model based elite income and elite numbers in [2].

I use empirical proxies for elite income and elite numbers to test one central prediction of the structural-demographic theory: as relative wages fall, elite incomes display a hump-shaped pattern while elite numbers increase. Importantly, the decline in elite incomes is a prediction of the theory and not the result of a particular model implementation.

**Fig 2:**
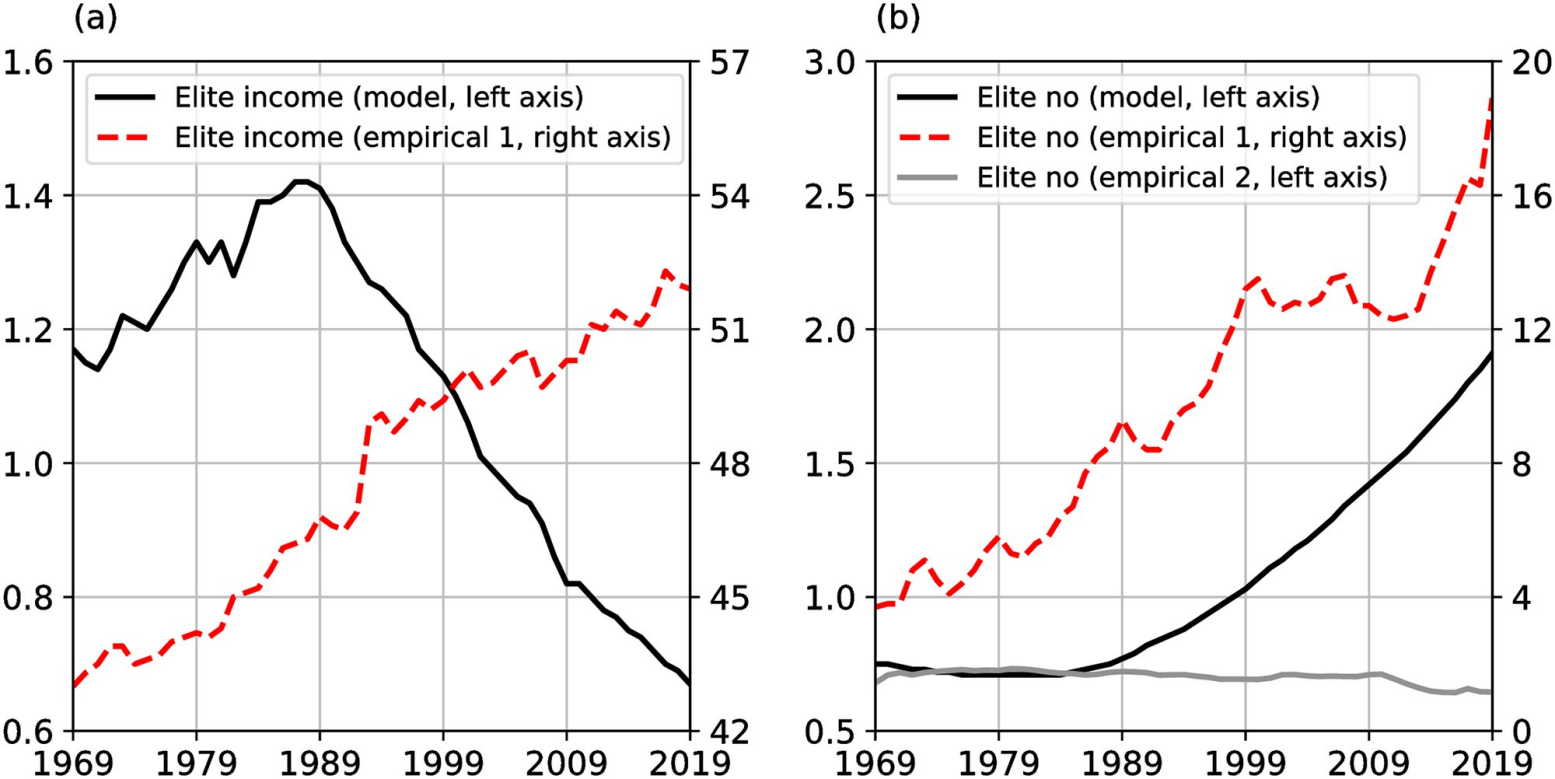
Elite numbers and income. (a) Model elite income as reported in [2], (black line). Empirical elite income is the share of the national income earned by households with incomes larger than the 80^*th*^ percentile of the US household income distribution (red dashed line). (b) Model elite numbers as reported in [2] (black line). Elite no empirical 1 is the share of households with income larger than $150,000 expressed in 2020 dollars (red dashed line). Elite no empirical 2 is the number of students enrolled in US law schools per 10,000 US inhabitants (grey line).

Panel (a) in Fig 2 below compares the model implied elite income, *e*, as reported in [2] (black line) with the elite income based on an empirical estimate (dashed red line). The empirical elite income is defined as the share of national income of households with incomes larger than the 80^*th*^ percentile of the income distribution. Panel (b) compares model implied elite numbers, *ϵ*, as reported in [2] (black line) with two empirical proxies of elite numbers. The first proxy is defined as the share of households with income larger than $150,000 expressed in 2020 dollars (red dashed line). The second proxy for elite numbers is defined as the number of students enrolled in US law schools per 10,000 US inhabitants (grey line).

Two observations emerge from the above comparison. First, while model implied elite income peaks around 1987 and falls steeply after, the empirical elite income rises steadily over the sample period. Second, the pattern of the elite numbers depends on the proxy variable used. While the household survey measure is increasing over the last three decades, consistent with the model implied elite numbers, the proxy variable based on enrolled law students hovers around the same level until 2009 and decreases thereafter. These data cast doubt on the external validity of the elite variable proxies used by [2]. Moreover, the pattern of elite incomes is not consistent with the predictions made by the theory: as relative wages fall, elite incomes increase, in contrast to the hump-shaped pattern displayed by the model based elite income in S2 Fig.

The argument that labor oversupply depresses wages leading to elite overproduction is not consistent with empirical data discussed above: elite incomes seem to increase in line with elite numbers (see Fig 2). The steady increase in elite incomes observed for the first empirical proxy of elite numbers is consistent with the empirical studies showing that the supply of college educated workers and the college wage premium increased in tandem in the US starting in the 1980s [12]. This trend is further confirmed by the monotonous increase in the return to education throughout most of the 20^*th*^ century, in particular in the last three decades (see [9, 21, 22]). In this context, the increase in tuition fees for undergraduate and graduate education need not be interpreted as evidence of elite overproduction (see [1], p. 204) but rather as a consequence of the increase in the demand for skills. Indeed, demand for skills exceeded supply for most of the period starting from 1850 to the present, widening the gap between the wages of skilled and unskilled workers and increasing inequality [23, 24].The evidence discussed in this section suggests that the second prediction of the structural-demographic theory for industrialized societies is not supported by the data.

## Instability principle

**Fig 3:**
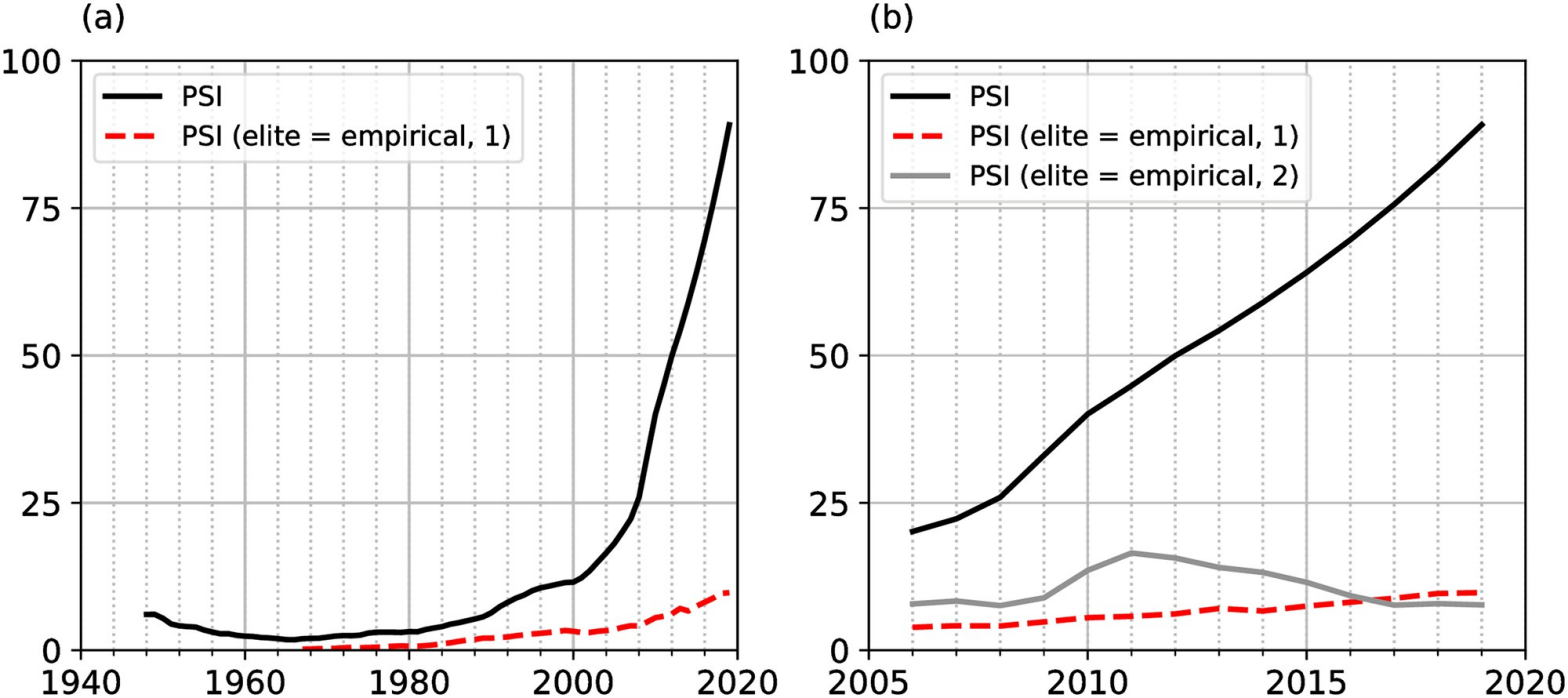
PSI using different elite measures. (a) and (b) The black line shows the PSI as implied by the model in [2]. The dashed red line shows the PSI computed using the elite variables based on the US Census household data. (b) The grey line shows the PSI computed using the elite variables based on US law school students’ data.

Political instability is quantified by the Political Stress Index as in [2]. The mass mobilisation potential and the elite mobilisation potential are used as inputs for the derivation of the Political Stress Indicator. To see to what extent model predictions are affected by the dynamics of elite income and elite numbers, Fig 3 plots the Political Stress Indicator (PSI) using the empirical proxies of these variables. The dashed red line in both panels shows the PSI computed using the elite numbers and elite income sourced from the US Census household data. The grey line in panel (b) shows the PSI computed using the elite numbers based on the number of enrolled law students and the elite income derived from law school graduates’ income.

The difference between the model implied and empirical PSI evolution is striking. While the model implied PSI experiences a steep increase in the period starting from 2010 onward, the PSI based on household survey data stays flat throughout the sample period, as rising elite incomes outweigh the impact of rising elite numbers on the PSI. The PSI based on lawyers’ data is only plotted starting 2006, due to the short time series available for lawyer’s income. The resulting path of the PSI is mostly flat throughout the sample period with a peak in 2010. These results imply that the upward path of the PSI reported in [2] is the result of a particular modelling choice. Thus, I find no evidence for the third prediction generated by the structural-demographic theory: elite overproduction did not cause a steep increase in the US Political Stress Indicator over the period 1960 to 2020. It follows that elite overproduction did not predict political instability in the US over that period.

The upward trend in the model implied PSI in the last decades has been compounded by rising public debt and distrust in the government, see S8 Fig. Considering the global perception of US public debt as safe haven, it seems unlikely that the steep increase in government debt is a symptom of financial or political fragility fuelling social unrest [25]. Consistent with this reasoning, the cost of US government debt has been on a steady downward path since 1980. While high public debt can be perceived as a source of concern by the public, concern about rising public debt does not move in tandem with public debt. In 2013, 72% of surveyed Americans considered that reducing the federal budget should be a top priority. The share has been falling steadily thereafter, reaching 48% in 2019 [26].

The increase in the model implied PSI correlates with the rise in political instability in the US in the period between 2010 and 2019. This correlation is interpreted as evidence supporting the predictions of the structural-demographic theory. Nevertheless, the model variables—relative wage, government debt, the share of the population aged between 20 and 29 and distrust in the government—explain only around 18% of the variance in political instability (see S1 Table). When correctly specified, models with such low explanatory power can shed light on the relation between the variables of interest. However, they are of limited usefulness for conducting out-of-sample forecasts. In other words, the model can be used for understanding rather than forecasting. The low external validity of the elite variables used as input for the derivation of the PSI in combination with the low-explanatory power of the model variables suggests that the predictions of the structural-demographic theory regarding political instability need to be interpreted with caution.

## Discussion

In this study, I test three predictions generated by the structural demographic theory for industrialized societies on US data. These predictions are not supported by the evidence discussed in the previous sections.

First, labor oversupply does not explain the variance of wages and the decline in relative wages. Crucially, relative wages are essentially a measure of income inequality, therefore the drop in relative wages needs to be interpreted in conjunction with the determinants of inequality. The stagnation and polarization in real wages, and the resulting decrease in relative wages is explained mostly by automation, followed by globalization and to a lesser extent by the erosion of labor market institutions. Second, the pattern of empirical elite incomes is not consistent with the predictions made by the theory: as relative wages fall, elite incomes increase, in contrast to the hump-shaped pattern displayed by the model based elite income. Importantly, the pattern of the elite numbers depends on the proxy variable used. Third, I find no empirical evidence for the prediction that elite overproduction predicted political instability in the US over the last decades. The increase in the Political Stress Indicator in the US in the last decades is driven by the increase in inequality. As mentioned above, the increase in inequality was mainly caused by technological change, followed by globalisation and eroding labor market institutions.

The hypothesis that the increase in inequality may be an important factor explaining the recent spike in social unrest is consistent with the cross-country evidence for a sample of 71 countries showing that higher inequality leads to political instability (see [27]). However, the low-explanatory power of the model variables in predicting political instability in the US in conjunction with the low external validity of the elite variables suggests that more research is needed to understand the drivers of the increase in political instability in the US in the last decade.

Attributing the rise in inequality and the resulting increase in political instability to labor oversupply rather than to the lost race between education and technology may weaken incentives to design effective policies addressing the inefficiencies in the US education system. For instance, the predictions of the structural-demographic theory are invoked to suggest that there is an “overproduction of young graduates with advanced degrees” in the United States and that “we should not expand our system of higher education beyond the ability of the economy to absorb university graduates. An excess of young people with advanced degrees has been one of the chief causes of instability in the past.” [28] This recommendation is at odds with the evidence discussed in the first section showing that demand for skills has increased in the last decades. Policymakers may be better advised to expand access to education to curb inequality, rather than to cap the supply of graduates.

Similarly, attributing the steep rise in inequality at the very top of the income distribution to elite fragmentation and increased intra-elite competition diverts attention from the legal framework that facilitates this outcome. For instance, since 1994, a change in the US tax code allowed firms to deduct top executive pay in excess of USD 1 million to the extent that it was linked to performance. As a result, CEO pay was linked to short-term performance measures such as earnings per share, providing incentives for short-term rent-seeking behavior (see [29]). Finally, disregarding the impact that more automation—foreshadowed by the tremendous progress of AI research—will have on wages and future inequality may harm the most impacted demographic groups. Interdisciplinary dialogue among historians, economists and sociologists can facilitate a better understanding of the relation between inequality, declining public trust and political instability in industrialized countries.

## Supporting information

S1 Fig US population structure by age and urbanization level. Data from [2].
(TIF)

S2 Fig Relative wage, elite income and elite numbers. Data from [2].
(TIF)

S3 Fig Relative wages and inequality. Source: Percentiles of pre-tax national income: World Inequality Database; Relative wage: Supplementary Material in [2]. Inequality increases when the share of national income held by the top 1 percent increases, while the share held by the bottom 50 percent decreases.
(TIF)

S4 Fig Evolution of median wage. Median wage: weekly real earnings in CPI Adjusted Dollars, obtained from the Federal Reserve Bank of St.Luis; median male wage: hourly wage obtained from the Economic Policy Institute, as in [1], p. 226. See: Median hourly wages.
(TIF)

S5 Fig Employment share by task type. Data obtained from Table 3a in [12]. The bars show the change in the employment share between 1959 and 2007 by task content. Task content is matched to occupations using the US Department of Labor’s Dictionary of Occupational Titles (DOT). The task categories follow the classification in [12]. Non-routine cognitive tasks refer to managerial, professional, and technical occupations; routine cognitive tasks refer to clerical, administrative and sales occupations; routine manual tasks include production and operative occupations; non-routine manual tasks refer to service occupations.
(TIF)

S6 Fig Wages by task content. Data obtained from Table 3.b in [12]. The bars show the log real wages relative to the 1959 mean by task content. Task content are matched to occupations using the US Department of Labor’s Dictionary of Occupational Titles (DOT).
(TIF)

S7 Fig Task content by education. Data obtained from Table 4 in [12]. The bars show the task content across educational groups as of 1980. Task content is matched to occupations using the US Department of Labor’s Dictionary of Occupational Titles (DOT). The task categories follow the classification in [12]. Non-routine cognitive tasks refer to managerial, professional, and technical occupations; routine cognitive tasks refer to clerical, administrative and sales occupations; routine manual tasks include production and operative occupations; non-routine manual tasks refer to service occupations.
(TIF)

S8 Fig Distrust in government and public debt. Data on distrust from [2]. Debt/GDP, Cost of debt: Federal Reserve Bank of St. Louis.
(TIF)

S1 Table Predicted PSI versus predicted demonstrations. Column (1) shows the coefficient estimates from a regression having the PSI as a dependent variable and relative wage, government debt, the share of the population aged between 20 and 29 as well as distrust in the government as predictors. Column (2) shows the coefficient estimates from the same regression having demonstrations as a dependent variable. The independent variables are log transformed and are defined as in [2]. Elite income and elite numbers were not included in the regressions reported in **S1 Table** due to almost perfect multicollinearity with the relative wage. *t* statistics in parentheses *, ** and *** corresponds to p values smaller than 0.05, 0.01 and 0.001 respectively.
(XLSX)
