# Supplementary figures and images for: The structural-demographic theory revisited: An empirical test for industrialized societies
Source: PLoS One. 2023 Nov 2;18(11):e0287912. doi: 10.1371/journal.pone.0287912 (PMC10621949; doi:10.1371/journal.pone.0287912)

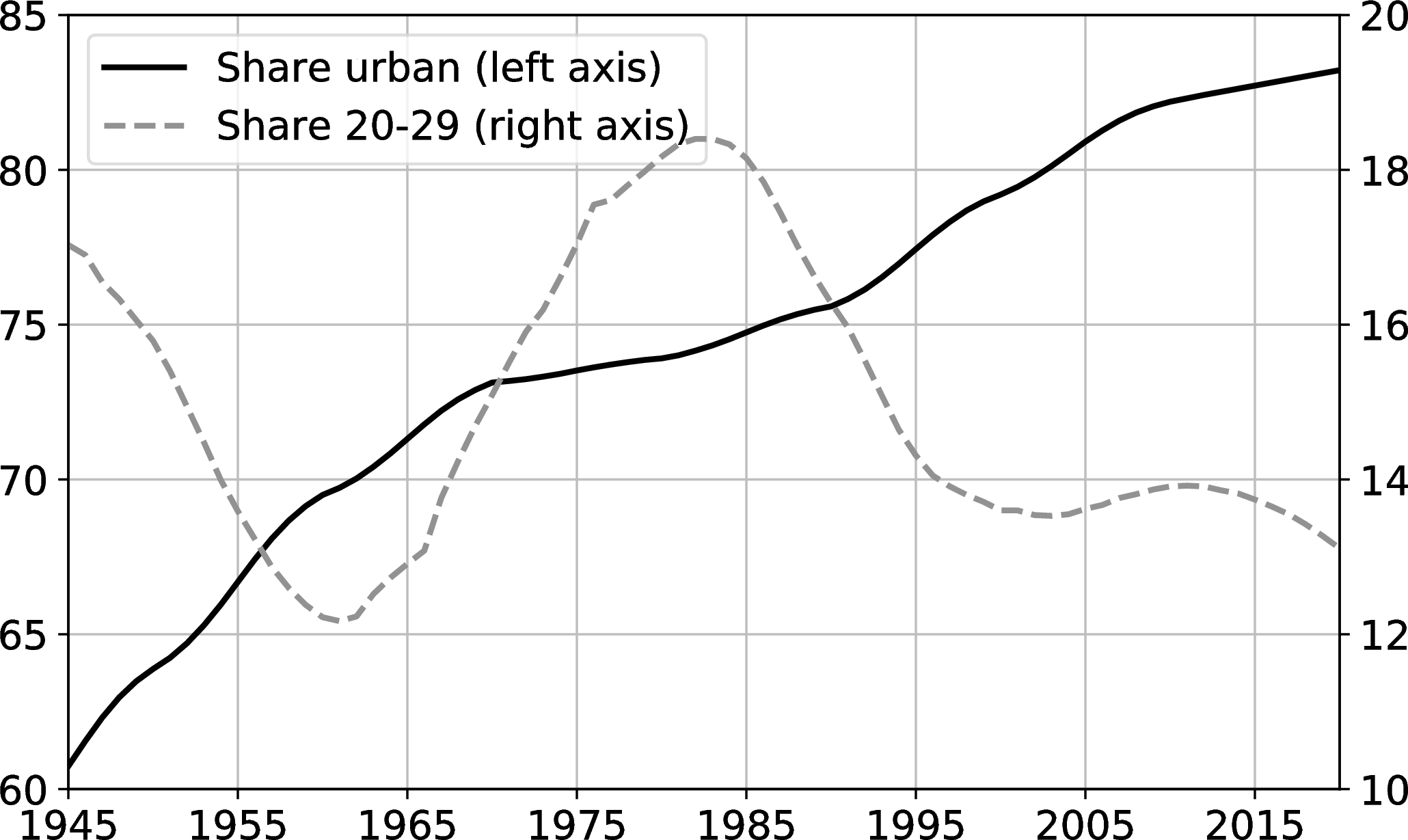

Supplement: S1 Fig — Data from [2]. (TIF) [file pone.0287912.s001.tif]

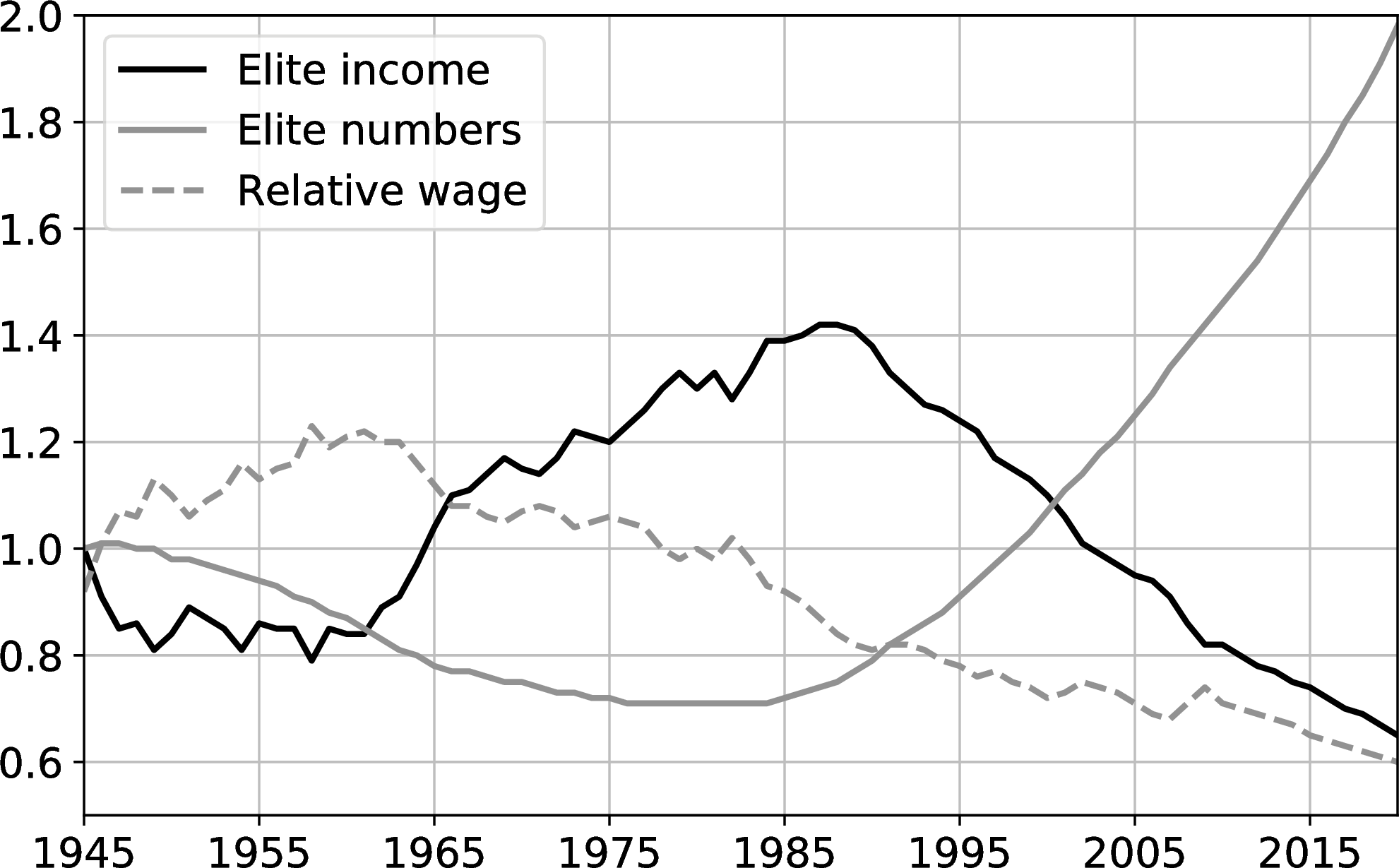

Supplement: S2 Fig — Data from [2]. (TIF) [file pone.0287912.s002.tif]

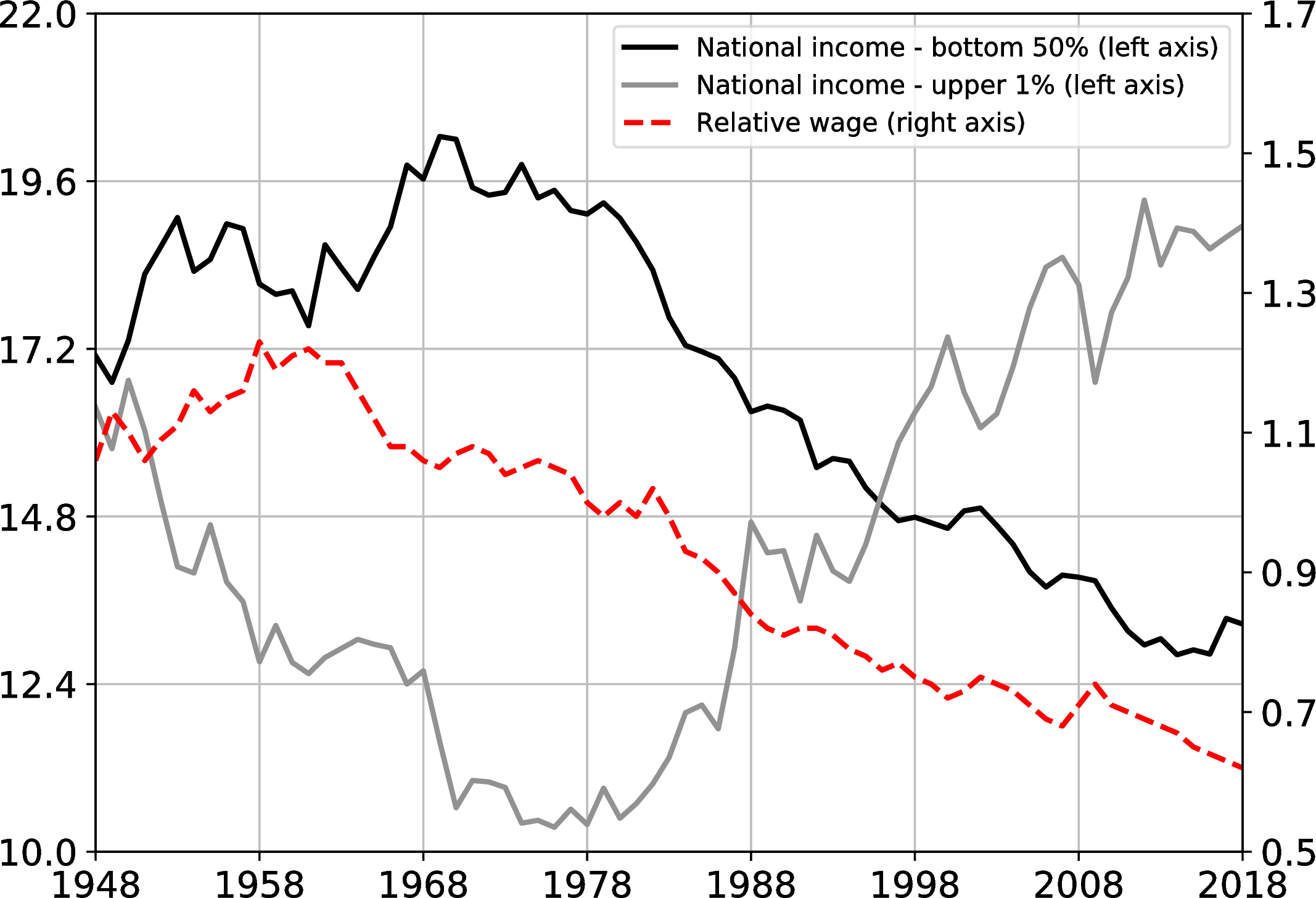

Supplement: S3 Fig — Source: Percentiles of pre-tax national income: World Inequality Database; Relative wage: Supplementary Material in [2]. Inequality increases when the share of national income held by the top 1 percent increases, while the share held by the bottom 50 percent decreases. (TIF) [file pone.0287912.s003.tif]

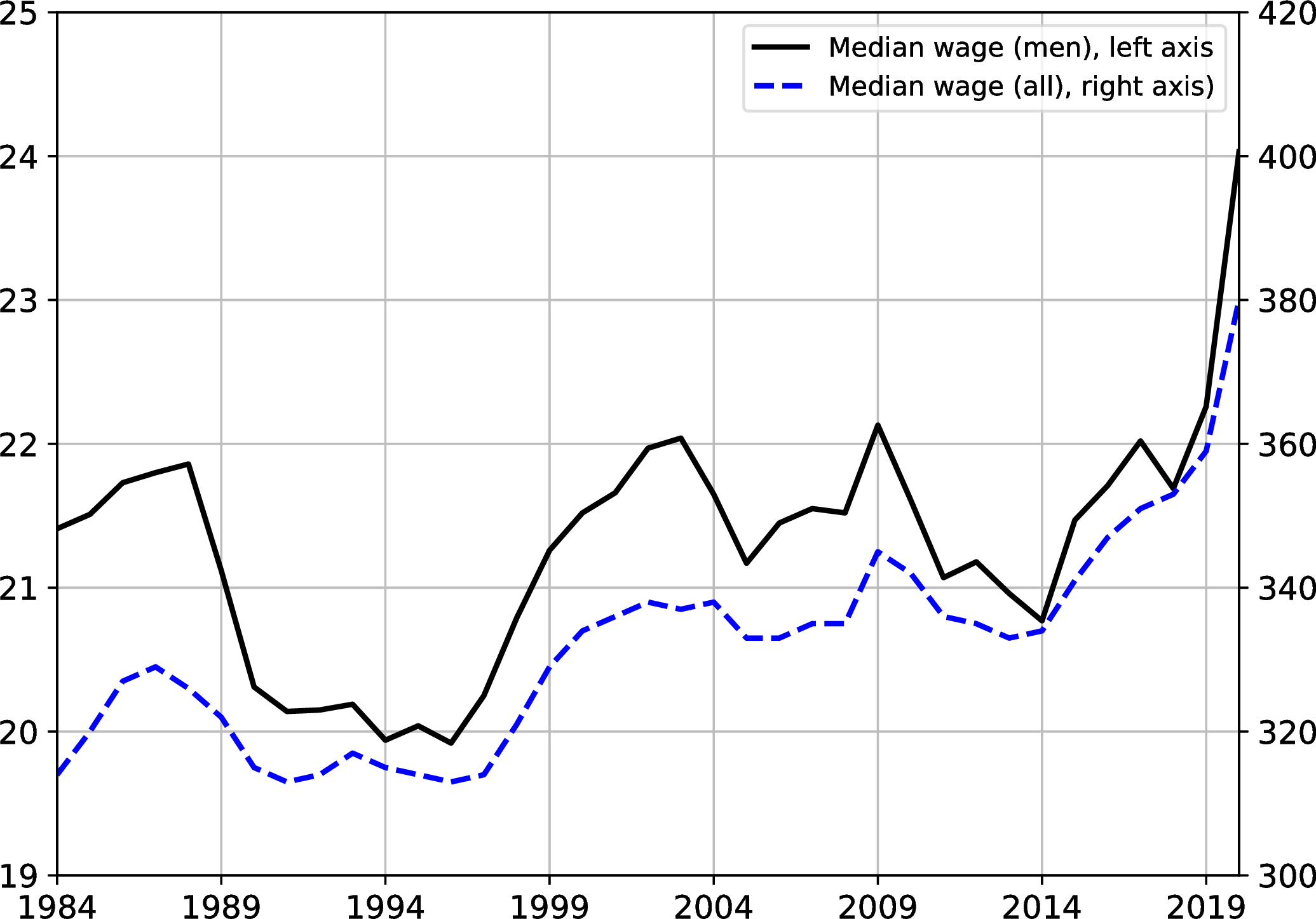

Supplement: S4 Fig — Median wage: weekly real earnings in CPI Adjusted Dollars, obtained from the Federal Reserve Bank of St.Luis; median male wage: hourly wage obtained from the Economic Policy Institute, as in [1], p. 226. See: Median hourly wages. (TIF) [file pone.0287912.s004.tif]

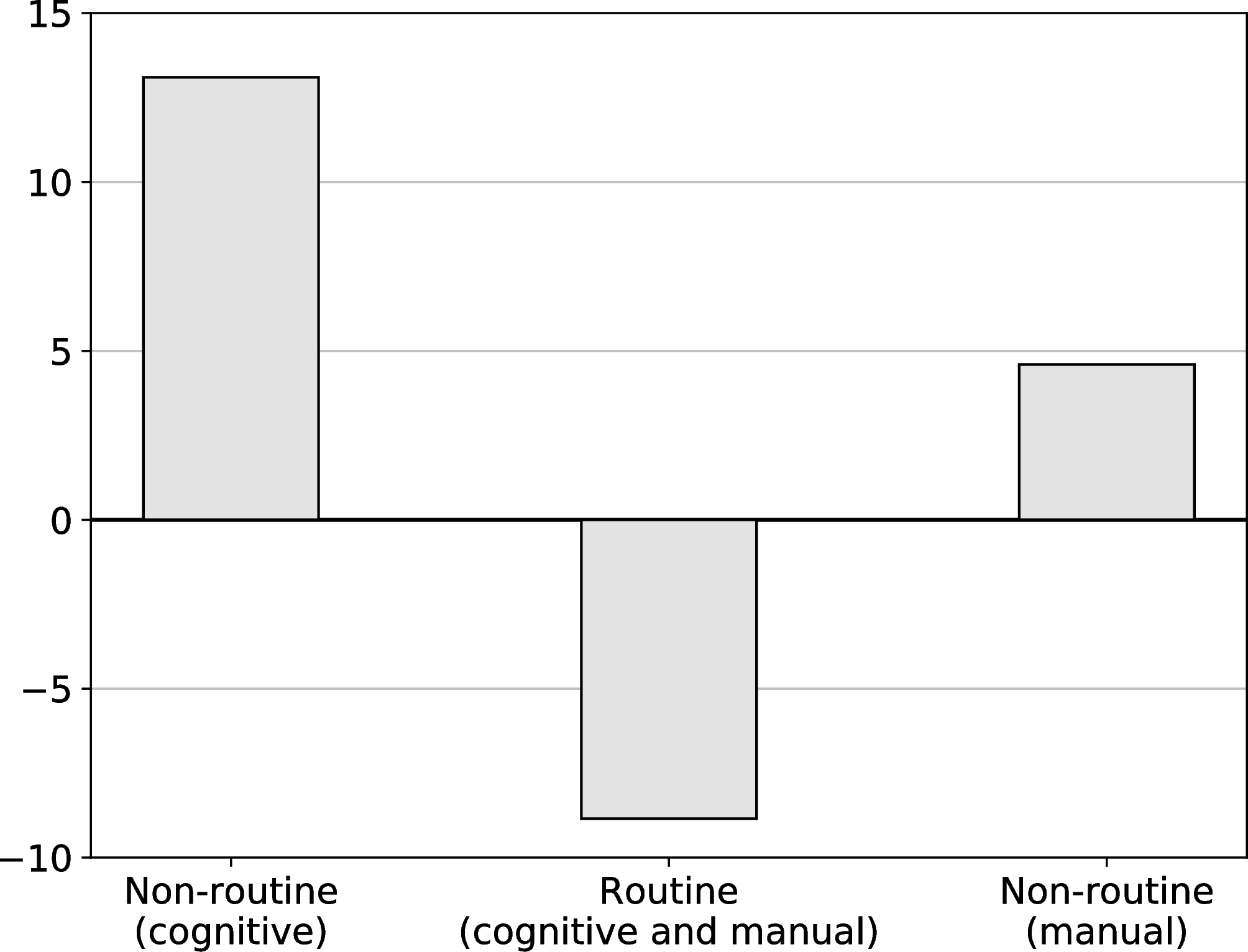

Supplement: S5 Fig — Data obtained from Table 3a in [12]. The bars show the change in the employment share between 1959 and 2007 by task content. Task content is matched to occupations using the US Department of Labor’s Dictionary of Occupational Titles (DOT). The task categories follow the classification in [12]. Non-routine cognitive tasks refer to managerial, professional, and technical occupations; routine cognitive tasks refer to clerical, administrative and sales occupations; routine manual tasks include production and operative occupations; non-routine manual tasks refer to service occupations. (TIF) [file pone.0287912.s005.tif]

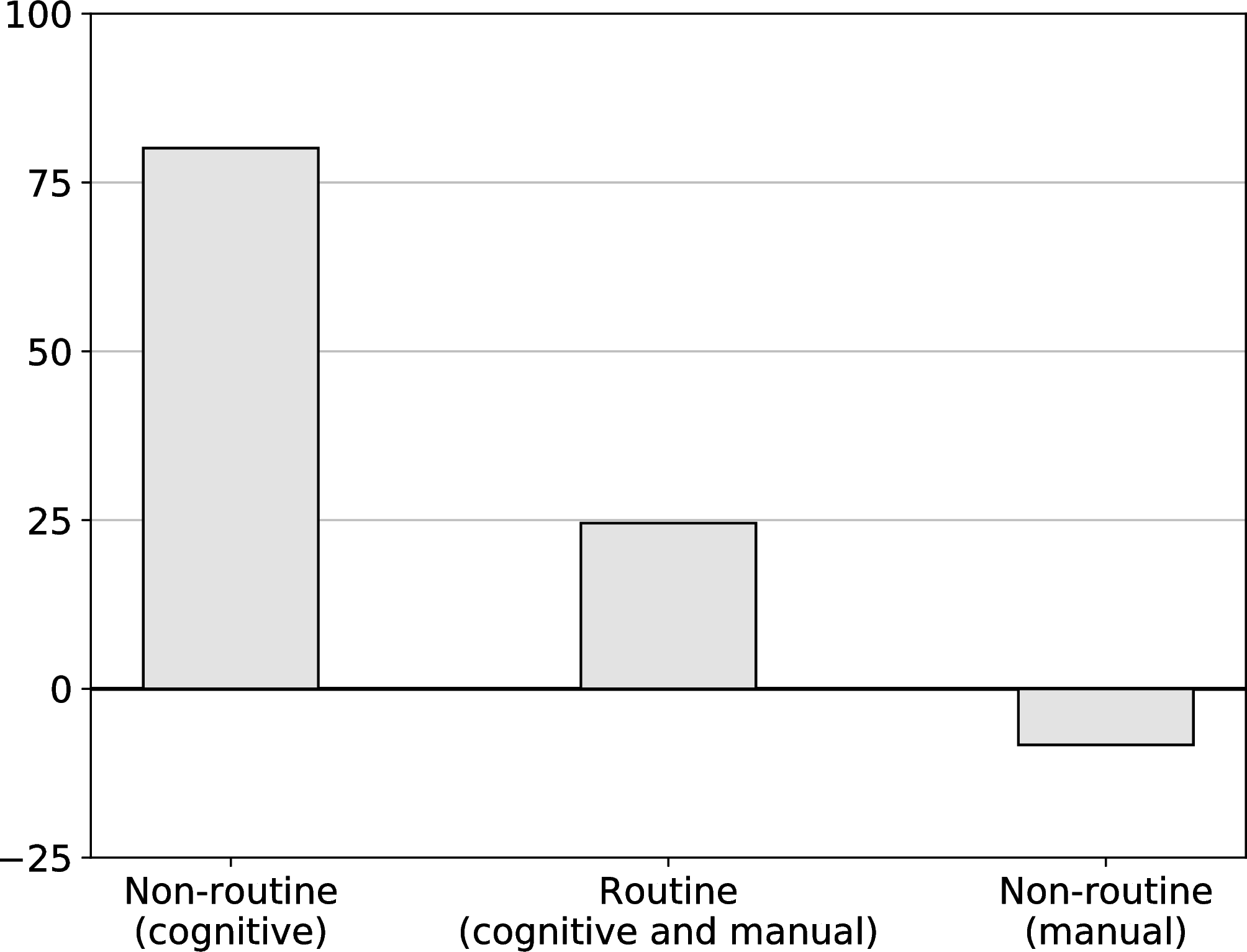

Supplement: S6 Fig — Data obtained from Table 3.b in [12]. The bars show the log real wages relative to the 1959 mean by task content. Task content are matched to occupations using the US Department of Labor’s Dictionary of Occupational Titles (DOT). (TIF) [file pone.0287912.s006.tif]

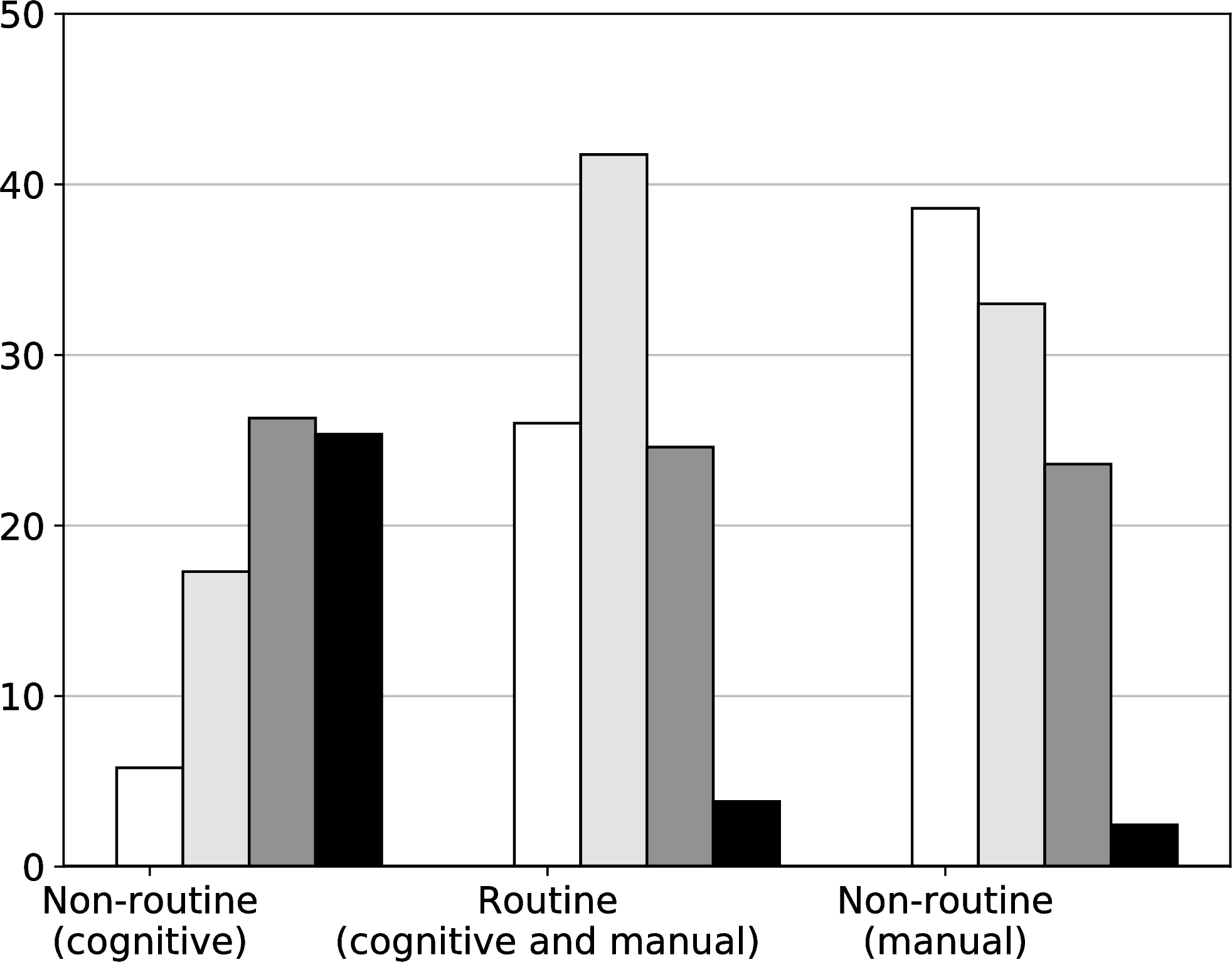

Supplement: S7 Fig — Data obtained from Table 4 in [12]. The bars show the task content across educational groups as of 1980. Task content is matched to occupations using the US Department of Labor’s Dictionary of Occupational Titles (DOT). The task categories follow the classification in [12]. Non-routine cognitive tasks refer to managerial, professional, and technical occupations; routine cognitive tasks refer to clerical, administrative and sales occupations; routine manual tasks include production and operative occupations; non-routine manual tasks refer to service occupations. (TIF) [file pone.0287912.s007.tif]

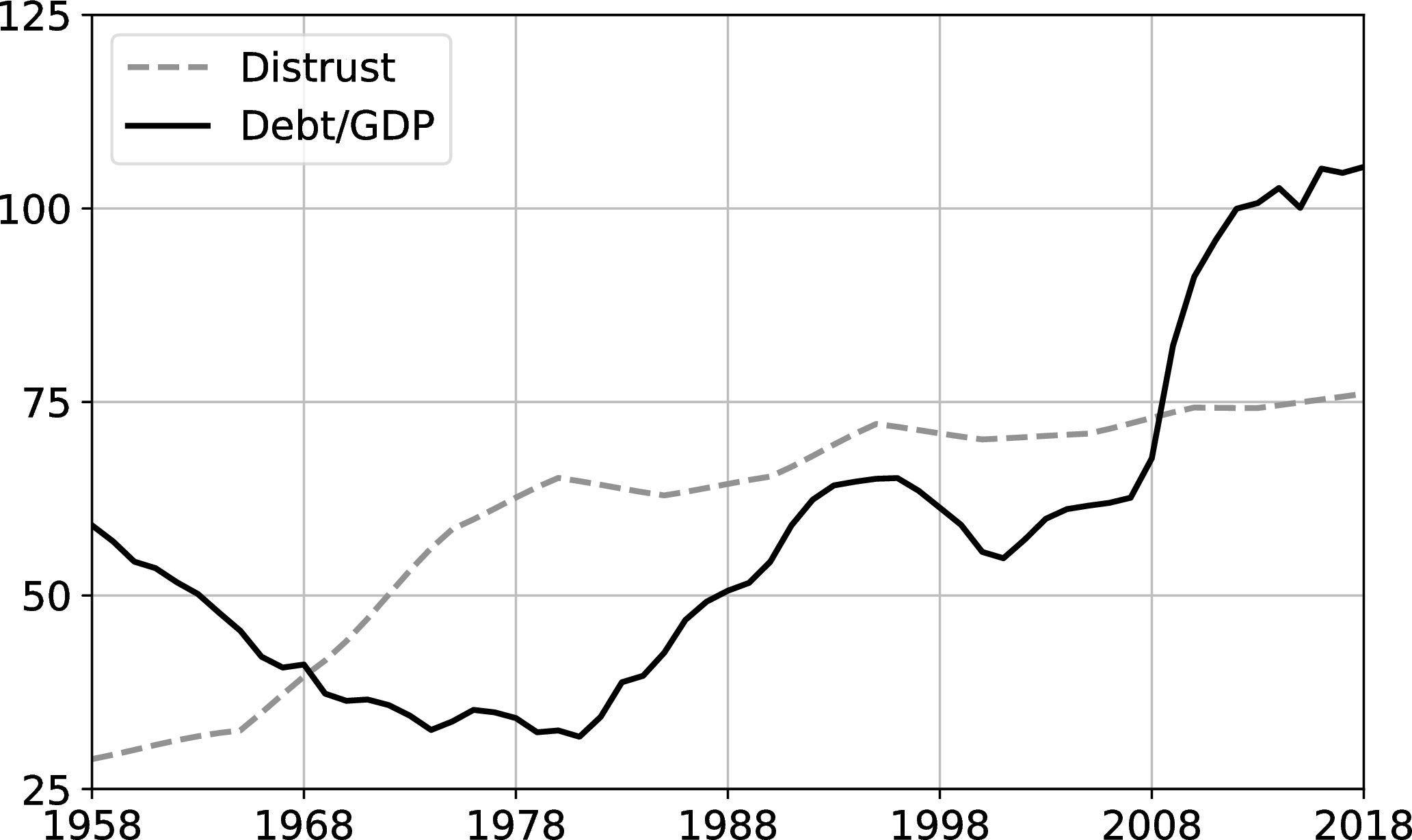

Supplement: S8 Fig — Data on distrust from [2]. Debt/GDP, Cost of debt: Federal Reserve Bank of St. Louis. (TIF) [file pone.0287912.s008.tif]
